# Chemoprotective effects of a recombinant protein from *Pyropia yezoensis* and synthetic peptide against acetaminophen-induced Chang liver cell death

**DOI:** 10.3892/ijmm.2015.2253

**Published:** 2015-06-19

**Authors:** YOUN HEE CHOI, EUN-YOUNG KIM, KOJI MIKAMI, TAEK JEONG NAM

**Affiliations:** 1 Institute of Fisheries Sciences, Pukyong National University, Busan 619-911, Republic of Korea; 2 Faculty of Fisheries Sciences, Hokkaido University, Hakodate 041-8611, Japan

**Keywords:** *Pyropia yezoensis*, recombinant protein, chemo protective effect, acetaminophen-induced cell death

## Abstract

In the present study, the chemoprotective effects of recombinant *Pyropia yezoensis* (*P. yezoensis*) protein 1 (PYP1) were examined in acetaminophen (APAP)-treated Chang liver cells. The analysis of *P. yezoensis* revealed the presence of both mature and immature variants of PYP1. PYP1s, designated as PYP1 (15 kDa), PYP1-AC (12 kDa) and PYP1-B (5 kDa), were successfully expressed in *Escherichia coli*, and their chemoprotective effects were then examined. In addition, a peptide of 11 residues (ALEGGKSSGGG), which is a common sequence at the N-terminus all of the PYP1s, was synthesized and examined. The effects of treatment with PYP1s and the synthetic peptide (SP) on cell proliferation were determined by MTS assay. Our results clearly demonstrated that treatment with all the PYP1s and SP significantly promoted the proliferation of Chang liver cells, protecting them against APAP. Thus, we concluded that recombinant PYP1s exert protective effects against injury to Chang liver cells.

## Introduction

Seaweed comprises abundant bioactive antioxidants, soluble dietary fibers, proteins, minerals, vitamins, phytochemicals and polyunsaturated fatty acids (1). It has been reported that these bioactive components (e.g., fucoxanthin, fucoidan and other extracts) exert a variety of effects in humans, such as anti-obesity (2), anticoagulant (3) and antitumor effects (4). The value of these properties as a function of bioactive substances in seaweed has been studied with regards to food, pharmaceuticals and medicinal purposes (1). In particular, *Hizikia fusiformis* extract was found to protect against injury to the liver and stomach in rats; this was proven by studying its molecular mechanisms of action (5,6). Moreover, it has been reported that a glycoprotein from *Pyropia yezoensis (P. yezoensis)* exerts anti-inflammatory effects in RAW 264.7 mouse macrophages (7). In addition, a protein isolated from *P. yezoensis* was shown to exert chemoprotective effects on acetaminophen (n-acetyl-p-aminophenol, APAP)-induced liver injury in rats (8). APAP is an over-the-counter drug that is widely used for its analgesic and antipyretic effects. APAP is safe when used at therapeutic levels; however, an acute or cumulative overdose can cause severe liver injury and possibly liver failure (9). Studies on APAP toxicity have been investigated both *in vitro* (10–13) and *in vivo* (8,14–17). The ability of seaweed to exert chemoprotective effects against APAP means they are advantageous to various organisms.

Recently, the association between the molecular structure and function of seaweed was reported (18). Choi *et al* (18) reported that the synthetic peptide (SP) ALEGGKSSGGGEATRDPEPT, which is present at the N-terminus of mature *P. yezoensis* protein 1 (PYP1), demonstrated chemoprotective effects against APAP-induced Chang liver cell death.

In the present study, 3 proteins (PYP1, PYP1-AC and PYP1-B) were derived from the cDNA that encodes PYP1 and were then investigated for their chemoprotective effects against APAP-induced Chang liver cell injury. Moreover, the N-terminal 11 residue sequence of SP, ALEGGKSSGGG, which represents a common sequence among all 3 peptides, was synthesized and compared with the PYP1s.

## Materials and methods

### Molecular cloning of cDNA and the gene encoding PYP1

To determine the N-terminal amino acid sequence of PYP1 (18), the *P. yezoensis* expressed sequence tag (EST) database of the Kazusa DNA institute (Chiba, Japan) was surveyed. Since the resultant information indicated that both mature and immature mRNAs were present, cDNA encoding these variants was cloned. Briefly, the cultivation of *P. yezoensis* gametophytes and the amplification of cDNA from total RNA were performed as previously described by Uji *et al* (19). DNA fragments corresponding to the open reading frames (ORFs) of mature and immature variants were then amplified by polymerase chain reaction (PCR) using the following primer sets: PYP1-F and PYP1-R, PYP1-F and PYP1-AC-R, and PYP1-F and PYP1-B-R (Table I). The PCR conditions were as follows: 30 cycles at 98°C for 10 sec and 68°C for 2 min using PrimeSTAR HS DNA polymerase with GC buffer (Takara Bio., Otsu, Japan). Separation, purification, cloning and sequence analysis were performed as previously described by Uji *et al* (19), with the exception of the pENTR/SD/D-TOPO vector (Invitrogen/Life Technologies, Carlsbad, CA, USA), which was used for the cloning and construction of plasmids that were expressed in bacteria as entry plasmids. To isolate the genomic fragment containing PYP ORF information, genomic DNA was prepared from gametophytes using a DNeasy Plant Mini kit (Qiagen, Hilden, Germany), and genomic PCR was performed as described above, using the PYP1-F and PYP1-R primers. The amplified fragment was inserted into a pCR-Blunt II-TOPO cloning kit (Clontech Laboratories, Inc., Mountain View, CA, USA) and sequenced.

### Expression analysis

To analyze the expression profile of the *PYP1* gene in both gametophytes and sporophytes of *P. yezoensis*, several generations were cultured and used for total RNA extraction in order to amplify the cDNA, as previously described by Uji *et al* (19). Following the synthesis of the first-strand cDNA using a PrimeScript II First Strand cDNA Synthesis kit (Takara Bio), reverse-transcription PCR (RT-PCR) was performed using Phusion High-Fidelity DNA polymerase (New England BioLabs, Inc., Beverley, MA, USA) with the primer sets described above, under the following conditions: 98°C for 1 min and 30 cycles at 98°C for 10 sec, 55°C for 30 sec, and 72°C for 1 min.

### Construction of PYP1 expression plasmids

Gateway Technology (Invitrogen/Life Technologies) was employed to construct the expression plasmids for the PYP1 and PYP1 variants in *Escherichia coli* (*E. coli*). To produce the destination vector, pQE-82L (Qiagen) was digested with *Sma*I in the multi-cloning site and ligated using Restriction fragment analysis (RFA; Invitrogen/Life Technologies). The resultant plasmid was designated as pQE80L-DES. LR recombination reactions were then performed with entry plasmids and pQE82L-DES according to the manufacturer’s instructions, thereby, producing pQE82L-PYP1, pQE82L-PYP1-AC and pQE82L-PYP1-B.

### Overexpression and purification of recombinant PYP1, PYP1-AC and PYP1-B

Plasmids (pQE82L-PYP1, pQE82L- PYP1-AC and pQE82L-PYP1-B) were transformed into the *E. coli* strain DH5α and incubated on ice for 30 min. S.O.C medium (200 *µ*l; Invitrogen Life Technologies) was added followed by incubation for 1 h at 37°C. The mixture was then spread on a plate with LB medium containing 100 *µ*g/ml ampicillin and incubated for 16 h at 37°C. After confirming the transformation in *E. coli* DH5α cells, the plasmids were transformed into the *E. coli* strain BL21 (DE3) in a similar manner.

For the induction of the expression of PYP1, PYP1-AC and PYP1-B, LB medium (20 ml) containing 100 *µ*g/ml ampicillin was inoculated and grown at 37°C overnight. Cultures were inoculated into 1 ml of LB medium containing ampicillin (100 *µ*g/ml; 1:50) with fresh LB culture. The cultures were grown at 37°C until an optical density (OD)_600_ of 0.5 was reached. One milliliter of sample was taken immediately prior to induction, and expression was induced by the addition of isopropyl β-D-1-thiogalactopyranoside (IPTG) to a final concentration of 1 mM. The cells were harvested by centrifugation at 4,000 × g for 20 min, resuspended in binding buffer (20 mM Tris-HCl, pH 8.0) and lysed by ultrasonic disruption (Sonics & Materials Inc., Newtown, CT, USA). The sonicated extracts were then separated into soluble and insoluble fractions by centrifugation at 4,000 × g for 20 min at 4°C.

To purify the recombinant proteins, soluble fractions containing the PYP1s were loaded onto an Ni-NTA column (Qiagen) with Ni-NTA His•Bind Resin (Merck Millipore, Darmstadt, Germany) using lysis buffer (50 mM NaH_2_PO_4_·H_2_O, 300 mM NaCl and 10 mM imidazole). After complete loading, weakly bound proteins were removed with wash buffer (50 mM NaH_2_PO_4_, 300 mM NaCl and 20 mM imidazole). Proteins were subsequently eluted from a Ni-NTA spin kit (Qiagen) with elution buffer (50 mM NaH_2_PO_4_, 300 mM NaCl and 250 mM imidazole), according to the manufacturer’s instructions. The concentrations of the protein samples collected during purification and purified samples were measured using the BCA method using a BCA protein assay kit (Pierce Biotechnology, Rockford, IL, USA).

All protein samples were resolved by 17% sodium dodecyl sulfate polyacrylamide gel electrophoresis (SDS-PAGE). For N-terminal sequencing analysis, proteins were transferred onto polyvinylidene fluoride (PVDF) membranes (Millipore, Billerica, MA, USA) using an electrophoresis power supply (GE Healthcare Bio-Sciences AB, Uppsala, Sweden).

### Peptide synthesis

The N-terminal 11-residue sequence, which was a common sequence in all the PYP1s (ALEGGKSSGGG), was synthesized by Peptron (Daejeon, Korea). Purification of the SP was performed on a Shimadzu Prominence HPLC system and controlled using the software package Class-VP, 6.14 with a C18 column (Shiseido Capcell Pak; Shiseido, Tokyo, Japan) in 0.1% trifluoroacetic acid (TFA)/water and a gradient of 10–70% acetonitrile in 0.1% TFA, at a flow rate of 1 ml/min and ultraviolet (UV) detection at 220 nm. The molecular mass was confirmed at 918 kDa (it matched the sequence mass) using mass analysis (HP 1100 Series LC/MSD; Agilent Technologies, Inc., Santa Clara, CA, USA).

### Cell culture

The Chang liver cell line (HPV-18) was obtained from the American Type Culture Collection (ATCC, Rockville, MD, USA). The cells were cultured in minimum essential medium (MEM) supplemented with 10% fetal bovine serum (FBS; HyClone, Logan, UT, USA), 100 U/ml penicillin and 100 mg/ml streptomycin. The cultures were maintained in a humidified incubator at 37°C in 5% CO_2_. The medium was replaced every 2 days.

### Cell proliferation assay

The effects of PYP1, PYP1-AC, PYP1-B and SP treatment on cell proliferation in the cells treated with 15 mM APAP were colorimetrically determined by MTS assays using CellTiter 96 AQueous One Solution reagent (Promega, Madison, WI, USA). The cells were seeded in 96-well plates at a density of 1.5×10^5^ cells/well. Following incubation for 24 h, the attached cells were maintained in serum-free medium (SFM) for 6 h, and this was followed by treatment with PYP1, PYP1-AC, PYP1-B or SP (0–1,000 pg/ml) for an additional 24 h. The cells were then incubated with MTS solution at 37°C for 30 min, and the absorbance was measured at 490 nm using a microplate reader (BioTek Instruments, Inc., Winooski, VT, USA). The OD_490_ values of the control cells were designated as 100%.

### 4,6-Diamidio-2-phenylindole (DAPI) staining assay

The cells were washed twice with phosphate-buffered saline (PBS) and fixed with 4% paraformaldehyde. The fixed cells were incubated at 37°C for 20 min and washed twice with PBS. The cells were then stained with 1 *µ*g/ml DAPI and incubated at room temperature for 20 min in the dark. The stained cells were observed under a fluorescence microscope (ECLIPSE TS100-F; Nikon Corp., Tokyo, Japan).

### Statistical analysis

Data were evaluated by one-way analysis of variance (ANOVA) using the Statistical Package for the Social Sciences version 10.0 (SPSS, Inc., Chicago, IL, USA). Significant differences between means were identified using Duncan’s multiple range test (P<0.05). A P-value <0.05 was considered to indicate a statistically significant difference.

## Results

### Identification and characterization of multiple PYP1 mRNA transcripts

**Figure 1:**
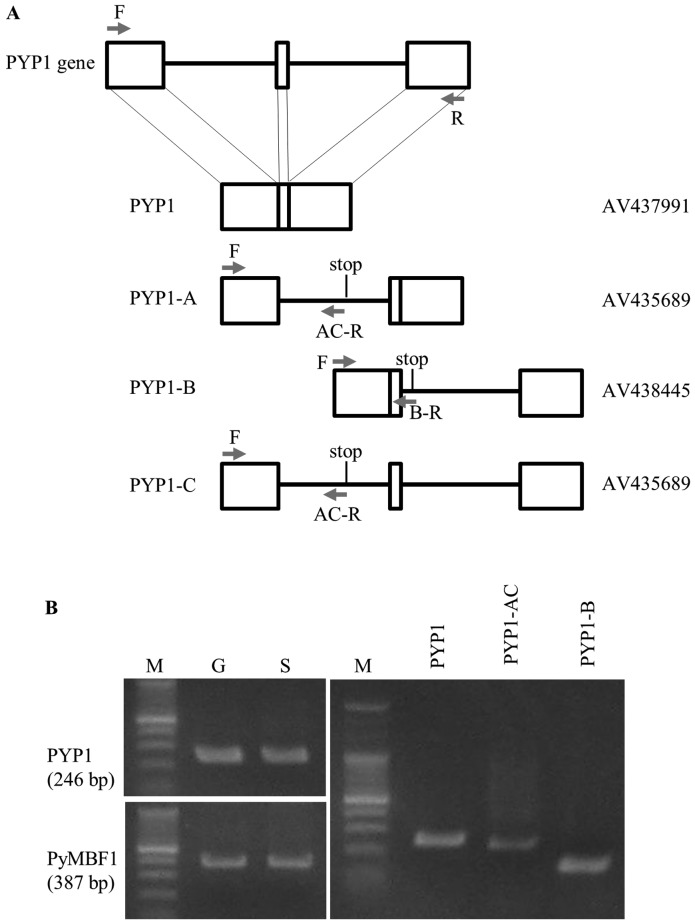
Identification and expression of mature and immature *Pyropia yezoensis* protein 1 (*PYP1*) gene transcripts. (A) Schematic representation of PYP1 and its variants produced by complete and alternative splicing, respectively, compared with the genomic organization of the *PYP1* gene. Empty boxes represent exons, while bars represent introns. Positions of stop codons are indicated in the schemes of each variant. AV numbers determined by the Kazusa DNA Research Institute are names of clones whose sequences were determined previously. Arrows indicate the positions of primers used for RT-PCR; (Table I): F, PYP1-F; R, PYP1-R; AC-R, PYP1-AC-R; B-R, PYP1-B-R. (B) Gel images showing the results from RT-PCR. Comparison of *PYP1* gene expression between gametophytic (G) and sporophytic (S) generations (left panel). The expression pattern of *PyMBF1* (19) is presented as a reference. The presence of transcripts from the mature form and splice variants in the gametophytic generation can be noted (right panel). The sizes of the RT-PCR fragments for PYP1, PYP1-AC and PYP1-B are 246, 232 and 141 bp, respectively. M, protein marker.

The SP, ALG EGKSSGGGEATRDPEPT, corresponding to the N-terminus of mature PYP1, has been shown to exhibit chemoprotective activity against APAP-induced Chang liver cell death (18). In order to obtain a full-length cDNA encoding PYP1 for use in inducing expression in bacteria, the *P. yezoensis* EST database of the Kazusa DNA Research Institute (http://est.kazusa.or.jp/en/plant/porphyra/EST/) was surveyed. A comparison of nucleotide sequences from the genomic *PYP1* gene revealed the presence of non-spliced introns, which were determined to cause the various lengths of *PYP1* mRNAs (Fig. 1A), indicating that alterative splicing produces mature and immature *PYP1* mRNAs.

**Figure 2:**
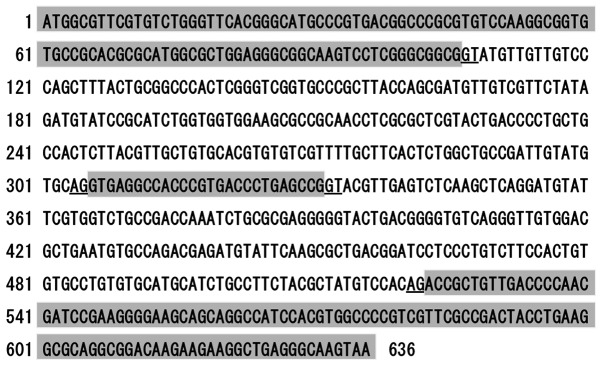
Exon-intron structure of *Pyropia yezoensis* protein 1 (*PYP1*) gene. The nucleotide sequence of *PYP1* is presented, and exons are highlighted. Exon-intron junctions fit the GT-AG rule.

As shown in Fig. 1A, the *PYP1* gene contains 2 introns; thus, the coding region is divided into 3 exons. Three types of immature cDNA contain either the first or second intron, or both introns. Proteins derived from the cDNA containing the first, second, or both introns were designated as PYP1-A, PYP1-B and PYP1-C, respectively (Fig. 1A). PYP1-A and PYP1-C encode the same protein, as they both contain the first intron, resulting in 3 PYP1 proteins: one is mature and the other two are variants, but all share an amino acid sequence corresponding to the first exon (Fig. 2). We refer to this mixture of PYP1-A and PYP1-C as PYP1-AC.

RT-PCR was employed to examine the expression of the *PYP1* gene in gametophytic and sporophytic generations of *P. yezoensis*. Since a homology search of *P. yezoensis* in the EST database resulted in ESTs derived from mRNAs of the gametophytic generation being found (data not shown), the generation-specific expression of the *PYP1* gene was speculated. However, Fig. 1B (left panels) clearly illustrates the expression of the *PYP1* gene in the gametophytic and sporophytic generations. Moreover, the presence of transcripts corresponding to PYP1-AC and PYP1-B was confirmed by RT-PCR (Fig. 1B, right panel), thus indicating that *P. yezoensis*-treated cells may contain all 3 PYP1 proteins.

### Preparation of bacterially expressed PYP1 proteins

To evaluate the functional activities of the 3 PYP1 proteins, PYP1, PYP1-AC and PYP1-B plasmids for the induction of expression in bacteria were constructed, which produced proteins with a 6xHis tag.

### Expression and purification of PYP1, PYP1-AC and PYP1-B

**Figure 3:**
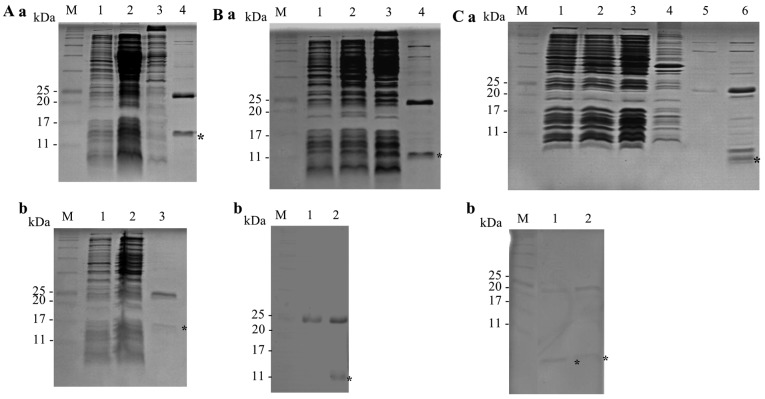
SDS-PAGE analysis of recombinant PYP1s expressed in the *E. coli* strain BL21. Samples were resolved by 17% SDS-PAGE. Recombinant proteins are indicated by an asterisk. (A) Recombinant PYP1. (a) M, protein marker; lane 1, before IPTG induction; lane 2, after IPTG induction; lane 3, insoluble protein; lane 4, first purified, soluble protein; (b) M, protein marker; lane 1, before IPTG induction; lane 2, after IPTG induction; lane 3, second purified, soluble protein. (B) Recombinant PYP1-AC. (a) M, protein marker; lane 1, before IPTG induction; lane 2, after IPTG induction; lane 3; soluble protein; lane 4, first purified, soluble protein; (b) M, protein marker; lane 1, control vector; lane 2, second purified, soluble protein. (C) Recombinant PYP1-B. (a) M, protein marker; lane 1, before IPTG induction; lane 2, after IPTG induction; lane 3, soluble protein; lane 4, insoluble protein; lane 5, first purified, soluble protein fraction 1; lane 6, first purified, soluble protein fraction 2; (b) M, protein marker; lanes 1 and 2, second purified, soluble protein. PYP1, *Pyropia yezoensis* protein 1.

The results of SDS-PAGE revealed purified soluble proteins (Fig. 3). PYP1, PYP1-AC and PYP1-B were first purified with Ni-NTA His•Bind Resin (Merck Millipore) (Fig. 3A-a, B-a and C-a). PYP1, PYP1-AC and PYP1-B were then further purified using a Ni-NTA Spin kit (Qiagen), which produced proteins with the following molecular weights: 15, 12 and 5 kDa, respectively (Fig. 3A-b, B-b and C-b). This two-step process used to extract PYP1s was effective.

### Physiological activity of PYP1, PYP1-AC and PYP1-B

**Figure 4:**
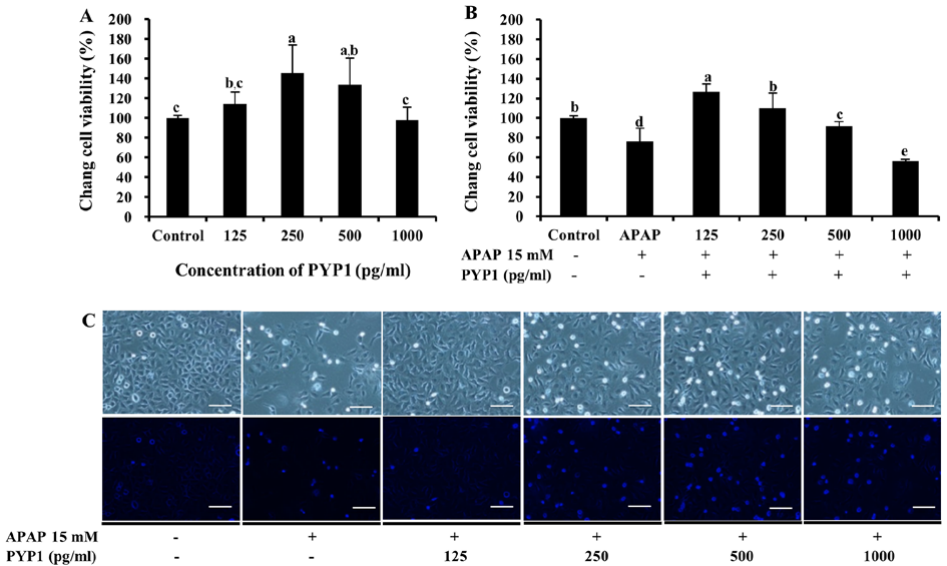
(A) Recombinant *Pyropia yezoensis* protein 1 (PYP1) toxicity in Chang liver cells. (B) Protective effects of PYP1 on acetaminophen (APAP)-induced liver damage. Cell viability was measured by MTS assays. Values represent the means ± standard deviation (SD) (P<0.05). Bars labeled with different letters indicate significant differences among groups, as determined by Duncan’s multiple-range test. (C) Morphological changes in cells following treatment with APAP alone or APAP + PYP1. Upper and lower panels show bright field and fluorescence images. Scale bar, 100 *µ*m.

**Figure 5:**
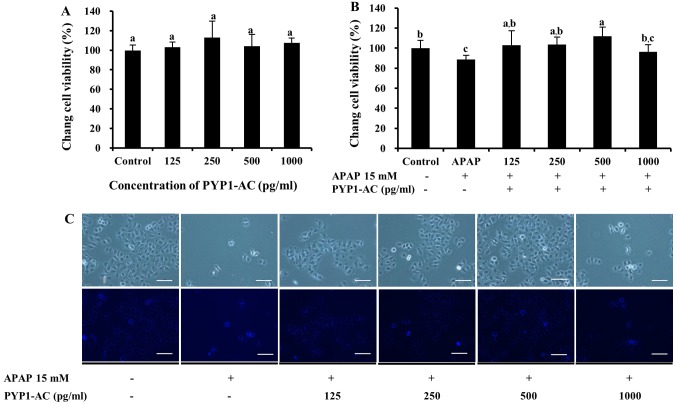
(A) Recombinant *Pyropia yezoensis* protein 1 (PYP1)-AC toxicity in Chang liver cells. (B) Protective effects of PYP1-AC on acetaminophen (APAP)-induced liver damage. Cell viability was measured by MTS assays. Values represent the means ± SD (P<0.05). Bars labeled with different letters indicate significant differences among groups, as determined by Duncan’s multiple-range test. (C) Morphological changes in cells following treatment with APAP alone or APAP + PYP1-AC. Upper and lower panels show bright field and fluorescence images. Scale bar, 100 *µ*m.

**Figure 6:**
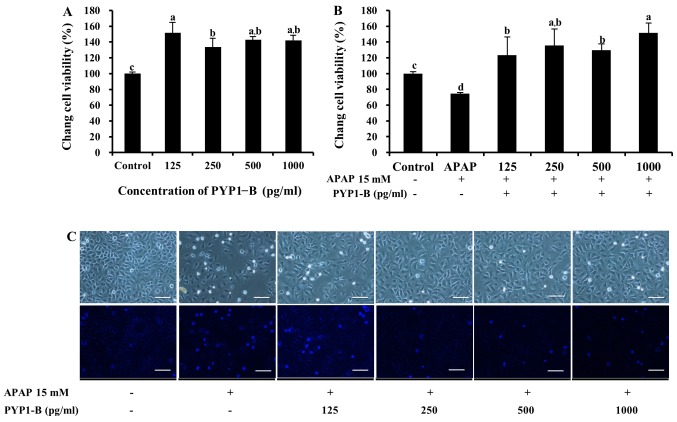
(A) Recombinant *Pyropia yezoensis* protein 1 (PYP1)-B toxicity in Chang liver cells. (B) Protective effects of PYP1-B on acetaminophen (APAP)-induced liver damage. Cell viability was measured by MTS assays. Values represent the means ± SD (P<0.05). Bars labeled with different letters indicate significant differences among groups,as determined by Duncan’s multiple-range test. (C) Morphological changes in cells following treatment with APAP alone or APAP + PYP1-B. Upper and lower panels show bright field and fluorescence images. Scale bar, 100 *µ*m.

The effects and toxicity of PYP1, PYP1-AC and PYP1-B on Chang liver cells were determined by MTS assays. As shown in Fig. 4A, PYP1 was non-toxic. Moreover, treatment with 125–500 pg/ml PYP1 increased cell viability from 114.2±12.5 to 145.0±28.2% (P<0.05) compared with the control. To determine whether PYP1 protects the Chang liver cells against APAP-induced death, cell viability was examined following treatment with APAP. The cells treated with 15 mM APAP demonstrated a viability of 76.6±13.3%. By contrast, when the cells were treated with 125–500 pg/ml PYP1, cell viability increased significantly to 127.0±7.8, 110.2±15.6 and 91.0±4.6% that of the control, respectively (Fig. 4B, P<0.05). The same result was noted in the cells stained with DAPI, according to fluorescence microscopy (Fig. 4C). Similar results were obtained for both PYP1-AC and PYP1-B (Figs. 5 and 6). No significant differences were observed with regard to cytotoxicity by PYP1-AC (P<0.05, Fig. 5A); however, cell viability increased significantly following treatment with 125–500 pg/ml PYP1-AC to 102.7±14.5, 103.5±7.6 and 111.7±9.3% compared to that of the control, respectively (P<0.05, Fig. 5B). These results are presented as photomicrographic images in Fig. 5C. Cell viability increased significantly to 151.4±13.6% following treatment with 125–1000 pg/ml PYP1-B (P<0.05, Fig. 6A). In addition, treatment with PYP1-B resulted in the highest value (151.4±12.6%) of all the APAP treatment groups (P<0.05, Fig. 6B). The effect of PYP1-B was also demonstrated by examining cellular morphology (Fig. 6C). These results suggest that recombinant PYP1s may be used to protect liver cells against APAP-induced cytotoxicity.

### Physiological activity of SP

**Figure 7:**
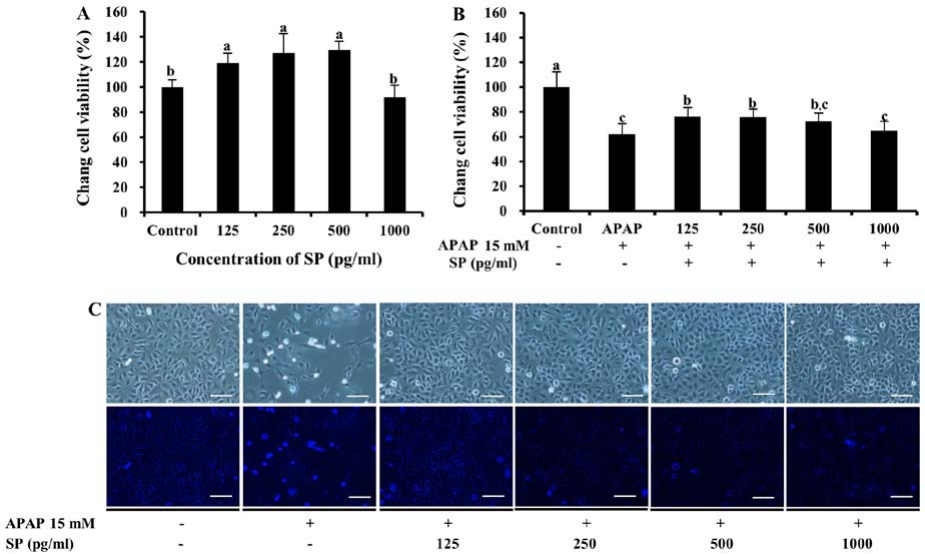
(A) Synthetic peptide (SP) toxicity in Chang liver cells. (B) Protective effects of SP on acetaminophen (APAP)-induced liver damage. Cell viability was measured by MTS assays. Values represent the means ± SD (P<0.05). Bars labeled with different letters indicate significant differences among groups, as determined by Duncan’s multiple-range test. (C) Morphological changes in cells following treatment with APAP alone or APAP + SP. Upper and lower panels show bright field and fluorescence images. Scale bar, 100 *µ*m.

To determine whether SP, which contains the N-terminal 11-residue sequence, protects Chang liver cells against APAP-induced death, cell viability was examined following treatment with APAP. As shown in Fig. 7A, SP was not toxic to the cells and resulted in cellular proliferation following treatment with 125–500 pg/ml SP. Notably, following treatment with both APAP and SP, cell viability decreased significantly compared with that of the control (P<0.05, Fig. 7B). Lower values upon SP treatment (64.9±7.5 to 75.9±7.5%, 125–1,000 pg/ml SP) compared to those observed with PYP1, PYP1-AC and PYP1 were observed in the APAP-treated cells. However, treatment with SP led to a significant increase in cell viability compared to treatment with APAP alone (61.8±8.6%; P<0.05, Fig. 7B). The cells treated with SP appeared to increase in number compared to the cells treated with APAP only (Fig. 7C).

## Discussion

In a previous study of ours (18), we suggested that PYP1, a novel protein from red alga *P. yezoensis*, had a sequence homology with that of hypothetical, unknown proteins from *Chondrus crispus* (Rhodophyta) and *Emiliania huxleyi* (Haptophyceae). The physicochemical characteristics of PYP1 are similar to those of late embryogenesis abundant (LEA) proteins. LEA proteins protect against protein denaturation caused by various environmental factors, such as desiccation, freezing, heat, salt and osmotic stress (20). Since *P. yezoensis* is one of the marine red algae that regulate environmental stress responses, it is frequently used to study molecular mechanisms (21).

Although APAP is an over-the-counter drug that is widely used for analgesic and antipyretic purposes, an overdose can cause liver injury, liver failure and even death (22). For these reasons, a variety of studies have investigated APAP metabolism in the liver, and various protective factors against APAP-induced liver toxicity, such as genistein (23) and manganese superoxide dismutase (SOD) (24), have been noted. Few researchers have studied the mechanisms of action of PYP since Hwang *et al* (8) reported the effects of PYP against APAP-induced liver cell injury. Currently, only one study using a particular PYP peptide has reported its regulation of multiple cell growth-related signals in MCF-7 cells (25). PYP has also been shown to stimulate the proliferation of IEC-6 normal intestinal epithelial cells and is associated with the insulin-like growth factor I (IGF-IR) and epidermal growth factor receptor (EGFR) signaling pathways (26,27).

In the present study, three PYP1 proteins and one SP containing the N-terminal 11-residue of the PYP1s were used to investigate the chemoprotective activities of recombinant PYP1s against APAP-induced cytotoxicity. We noted that SP, as well as PYP1 proteins, exhibited highly positive and supportive chemoprotective activities against APAP-induced toxicity. In addition, SP had a similar effect against methotrexate-induced cell death in Chang liver cells (data not shown). Therefore, these results suggest that the PYP peptide exerts protective effects against liver cell injury.

In general, macroalgae represent ideal starting materials for the generation of marine protein-derived bioactive peptides due to their high protein content (28). As these bioactive peptides act on numerous physiological functions (29), we should focus on macroalgae from natural materials. These peptides are involved in the modulation of cell proliferation-associated molecules (30). In addition, peptides from macroalgae have been reported to possess several biological traits, including ACE inhibitory and anti-hypertensive effects (31,32). Of note, Suetsuna and Saito (33) reported that PYP inhibits antimutagenesis and Ca^2+^ precipitation, lowers plasma and hepatic cholesterol, improves hepatic function, reduces blood sugar and exhibits antioxidant and SOD-like qualities; PYP also has angiotensin-converting-enzyme (ACE) inhibitory and anti-hypertensive effects. Our findings demonstrated that PYP1s, as bioactive peptides, have a positive effect against hepatic toxicity. However, the potential of this bioactive peptide *in vivo* remains to be elucidated; a specific biological activity observed *in vitro* is only an indicator of the potential active component *in vivo* (28). Therefore, further studies are required to address the biological activity of PYP1s *in vivo* and in signaling pathways.

## Figures and Tables

**Table I:** Primers used for PCR.

Primer	Sequence
PYP1-F	5′-CACCATGGCGTTCGTGTCTGGGTTCAC-3′
PYP1-R	5′-CTTGCCCTCAGCCTTCTTCTTG-3′
PYP1-AC-R	5′-GTACGAGCGCGAGGTTGCGG-3′
PYP1-B-R	5′-ACGTACCGGCTCAGGGTCAC-3′

PYP1 Pyropia yezoensis protein 1.
